# Temperature impacts SARS-CoV-2 spike fusogenicity and evolution

**DOI:** 10.1128/mbio.03360-23

**Published:** 2024-02-27

**Authors:** Jérémy Dufloo, Rafael Sanjuán

**Affiliations:** 1 Institute for Integrative Systems Biology, Consejo Superior de Investigaciones Científicas-Universitat de València, Paterna, València, Spain; University of Pittsburgh School of Medicine, Pittsburgh, Pennsylvania, USA; University of Illinois Urbana-Champaign, Urbana, Illinois, USA

**Keywords:** SARS-CoV-2 spike, temperature, experimental evolution, cell-to-cell fusion, SARS-CoV-2 variants

## Abstract

SARS-CoV-2 infects both the upper and lower respiratory tracts, which are characterized by different temperatures (33°C and 37°C, respectively). In addition, fever is a common COVID-19 symptom. SARS-CoV-2 has been shown to replicate more efficiently at low temperatures, but the effect of temperature on different viral proteins remains poorly understood. Here, we investigate how temperature affects the SARS-CoV-2 spike function and evolution. We first observed that increasing temperature from 33°C to 37°C or 39°C increased spike-mediated cell–cell fusion. We then experimentally evolved a recombinant vesicular stomatitis virus expressing the SARS-CoV-2 spike at these different temperatures. We found that spike-mediated cell–cell fusion was maintained during evolution at 39°C but was lost in a high proportion of viruses that evolved at 33°C or 37°C. Consistently, sequencing of the spikes evolved at 33°C or 37°C revealed the accumulation of mutations around the furin cleavage site, a region that determines cell–cell fusion, whereas this did not occur in spikes evolved at 39°C. Finally, using site-directed mutagenesis, we found that disruption of the furin cleavage site had a temperature-dependent effect on spike-induced cell–cell fusion and viral fitness. Our results suggest that variations in body temperature may affect the activity and diversification of the SARS-CoV-2 spike.

**IMPORTANCE:**

When it infects humans, SARS-CoV-2 is exposed to different temperatures (e.g., replication site and fever). Temperature has been shown to strongly impact SARS-CoV-2 replication, but how it affects the activity and evolution of the spike protein remains poorly understood. Here, we first show that high temperatures increase the SARS-CoV-2 spike fusogenicity. Then, we demonstrate that the evolution of the spike activity and variants depends on temperature. Finally, we show that the functional effect of specific spike mutations is temperature-dependent. Overall, our results suggest that temperature may be a factor influencing the activity and adaptation of the SARS-CoV-2 spike *in vivo*, which will help understanding viral tropism, pathogenesis, and evolution.

## INTRODUCTION

In humans, the severe acute respiratory syndrome 2 virus (SARS-CoV-2) mainly replicates in the upper and lower respiratory tracts. In SARS-CoV-2 patients, viral antigens have indeed been detected in the nasal epithelium, trachea, or lungs (1, 2). Moreover, under cell culture conditions, SARS-CoV-2 can infect nasal, bronchial, and lung primary human tissues (3). Infection can cause a wide range of symptoms, from completely asymptomatic infection to mild respiratory disease and even acute respiratory distress syndrome in the most severe COVID-19 cases (4).

The human airway is characterized by a temperature gradient from 25°C in the nasal cavity to 33°C in the pharynx and 37°C in the lungs. SARS-CoV-2 has previously been shown to replicate better at 33°C than at 37°C (5, 6). In addition to cough, sore throat, or fatigue, one of the most common symptoms associated with SARS-CoV-2 infection is fever. Hyperthermia has previously been shown to decrease SARS-CoV-2 replication *in vitro* (7). Temperature plays multiple roles in RNA virus transmission, replication, and antiviral immune responses (8). Low temperatures have been shown to favor spike interaction with its receptor ACE2 (9, 10), but the role of hyperthermia in viral entry is not well-characterized.

The SARS-CoV-2 spike protein is one of the three viral proteins exposed on the surface of viral particles, where it is present as an S1/S2 trimer. The S1 subunit mediates the interaction with its receptor ACE2, and S2 contains the fusion machinery necessary for fusion of the viral envelope with the target cell membrane. The SARS-CoV-2 spike possesses a multi-basic furin cleavage site (FCS) at the S1/S2 junction. This FCS allows cleavage and pre-activation of the spike in producer cells by furin. Upon interaction with ACE2 in target cells, a second proteolytic cleavage fully activates the spike. Depending on the target cell type, this is mediated either by TMPRSS2 at the plasma membrane or by cathepsins in the endosomes. The spike–ACE2 interaction not only mediates entry of viral particles but can also induce cell–cell fusion when the spike on the surface of an infected cell interacts with ACE2 expressed by a non-infected cell (11, 12). Such spike-mediated syncytia have been observed in COVID-19 deceased patients (13) and have been suggested to play a role in viral spread, pathogenesis, and immune escape (12). Spike-mediated syncytia formation depends on the presence of the FCS, as its deletion strongly decreases spike fusogenicity (14, 15). However, the effect of temperature on spike-mediated cell–cell fusion is currently unknown.

Since its emergence in humans in 2019, SARS-CoV-2 has evolved into subsequent lineages characterized by different sets of mutations across the whole viral genome. The spike protein is a highly variable region where some variants have accumulated more than 30 mutations. Spike mutations can alter ACE2 affinity, entry pathway, and transmissibility or confer antibody escape (16). Spike variants also differ in their ability to induce cell–cell fusion. For example, the Alpha, Beta, Gamma, and especially Delta variants were associated with increased syncytia formation compared to the ancestral Wuhan-Hu-1 strain (17). Conversely, the Omicron variants induce less cell–cell fusion (18–20). Moreover, spike mutations can alter the cellular and tissue tropism of SARS-CoV-2. For example, while ancestral strains mainly used TMPRSS2 to enter cells at the plasma membrane, the Omicron variants preferentially use the cathepsin-dependent endosomal entry pathway (18). This has been suggested to shift the tropism of Omicron from the lower to the upper respiratory tract. Accordingly, Omicron replicates better than ancestral strains in primary nasal and bronchial tissues at 33°C (3). Conversely, hyperthermia decreases Omicron replication to a greater extent than that of the Delta variant (21). Linking spike function, viral evolution, and temperature is therefore critical to understanding SARS-CoV-2 adaptation and pathogenesis.

Here, we investigate the effect of temperature on SARS-CoV-2 spike fusogenicity, and, using experimental evolution, we assess how temperature affects spike sequence diversification. We show that high temperatures increase spike-mediated syncytia formation. Moreover, we find that mutations that disrupt the FCS increase in frequency in spikes evolved at 33°C and 37°C, but not at 39°C, because the effects of FCS inactivation on spike-mediated cell–cell fusion, viral entry, and fitness are temperature-dependent. Taken together, these results suggest that temperature affects spike function and is one of the factors influencing the evolution of SARS-CoV-2.

## RESULTS

### High temperatures increase SARS-CoV-2 spike fusogenicity

**Fig 1:**
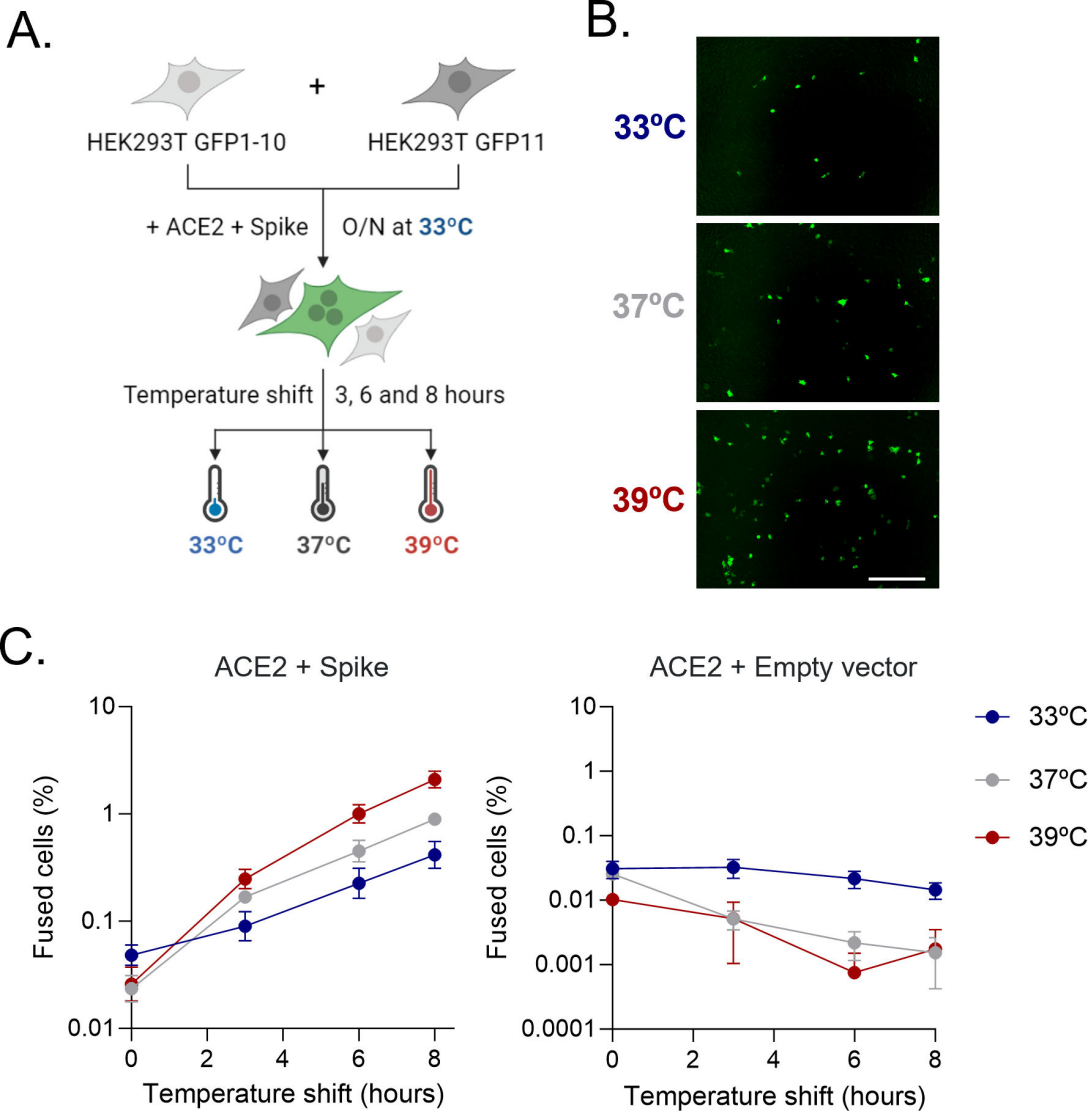
SARS-CoV-2 spike-mediated cell–cell fusion increases with temperature**.** (A) Schematic of the cell–cell fusion assay with temperature shift. HEK293T GFP–Split cells were mixed and transfected overnight at 33°C with ACE2 and the SARS-CoV-2 spike. Cells were then shifted to different temperatures (33°C, 37°C, and 39°C) for 3 h, 6 h, or 8 h, and GFP-positive syncytia were quantified. (**B)** Representative images of spike-mediated cell–cell fusion after 8 h at different temperatures. Scale bar: 1 mm. (**C)** Percentage of spike-mediated cell–cell fusion after shifting transfected GFP–Split cells to different temperatures. Data are shown for cells cotransfected with an ACE2-encoding plasmid and the SARS-CoV-2 spike plasmid (left panel) or an empty vector plasmid as a control (right panel). Mean and SEM are shown (*n* = 3).

To measure the effect of temperature on the fusogenicity of the SARS-CoV-2 spike, we used a previously described GFP complementation cell–cell fusion assay (11) (Fig. 1A). HEK293T cells expressing two different parts of GFP were mixed and transfected with the SARS-CoV-2 spike (Wuhan-Hu-1 strain) and ACE2. Cells were incubated at 33°C overnight and then transferred to different temperatures (33°C, 37°C, or 39°C) for 3 h, 6 h, or 8 h. The interaction between spike and ACE2 led to cell–cell fusion and reconstitution of GFP, which was measured by quantitative fluorescence microscopy (Fig. 1B). This showed that the kinetics of spike-mediated cell–cell fusion was temperature-dependent (Fig. 1C). The GFP signal observed after 8 h at 39°C was 2.4-fold higher than at 37°C and 4.8-fold higher than at 33°C. A general linear model of the effects of temperature, experimental block, and time (covariate) on the log GFP signal confirmed that temperature significantly increased spike-mediated membrane fusion over time (*P* < 0.0001). No cell–cell fusion was observed at any temperature in the absence of spike overexpression, ruling out a non-specific effect of temperature on cell–cell fusion (Fig. 1C).

### The evolution of spike fusogenic activity is temperature-dependent

**Fig 2:**
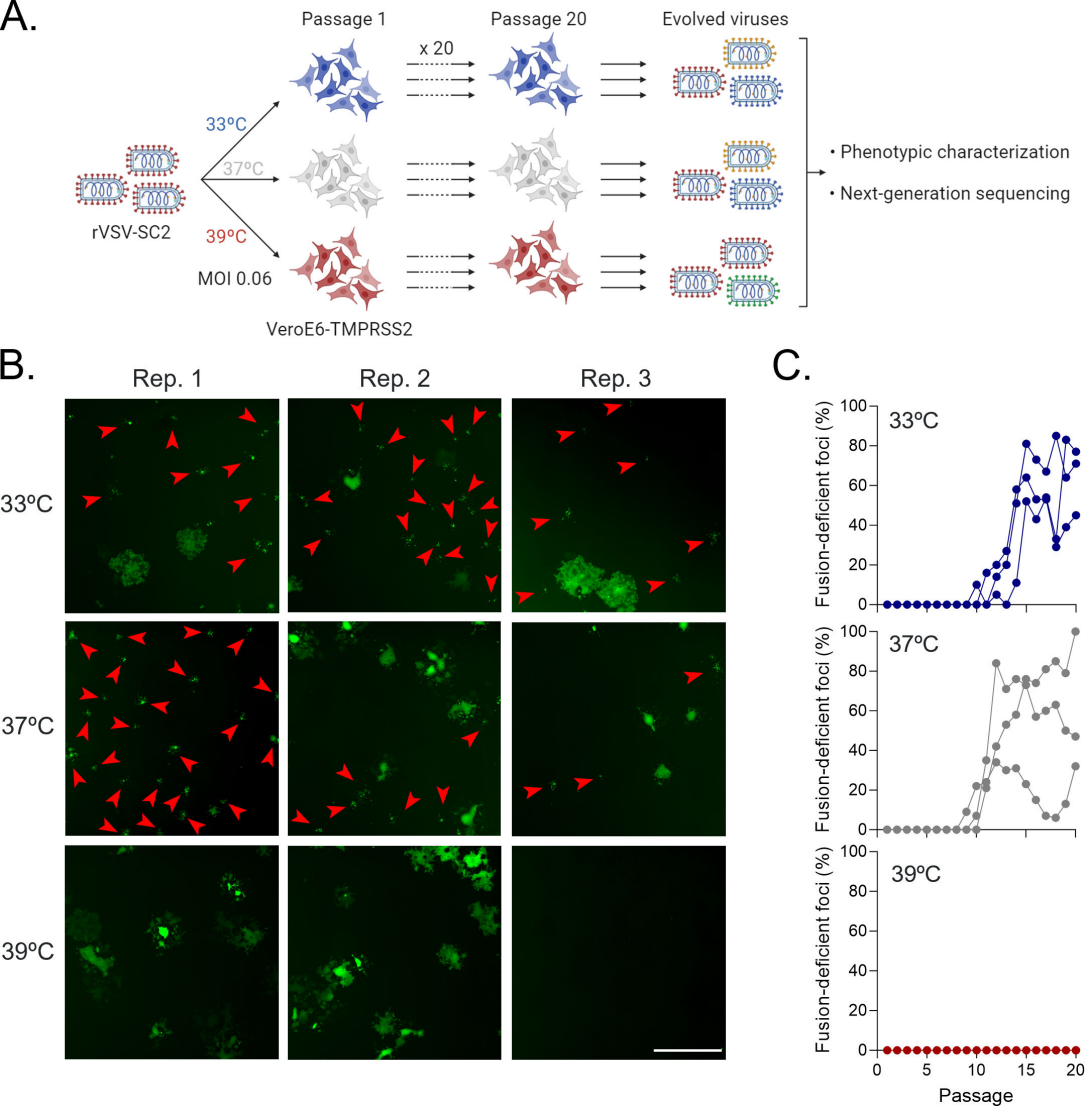
rVSV-SARS-CoV-2 phenotypic evolution is temperature-dependent. (A) Schematic of the experimental evolution. Recombinant VSV expressing the SARS-CoV-2 spike was passaged 20 times in VeroE6-TMPRSS2 at 33°C, 37°C, or 39°C. Three independent evolution lines were performed per temperature. Evolved viral populations were characterized phenotypically, and the spike gene was sequenced. (**B)** Representative images of the titration of viruses passaged 20 times in VeroE6-TMPRSS2 at different temperatures. Red arrowheads indicate foci of viruses with impaired cell–cell fusion activity. Scale bar: 1 mm. (**C)** Quantification of the percentage of fusion-deficient foci from the titration of each evolution passage. Each line represents an independent evolution replicate (*n* = 3).

We then investigated whether temperature affected spike diversification using an experimental evolution approach (Fig. 2A). We obtained a GFP-expressing recombinant vesicular stomatitis virus (VSV) modified to express the SARS-CoV-2 spike (strain Wuhan-Hu-1; rVSV-SC2) instead of the VSV G envelope glycoprotein. Using a recombinant VSV virus is a safe, relevant, and convenient way to study SARS-CoV-2 spike function and evolution. First, the activity (e.g., entry pathway and recognition by anti-spike antibodies) of the SARS-CoV-2 spike is similar between a VSV recombinant and a real SARS-CoV-2 virus (22–24). Second, previous work has already used this system to study the adaptation of the SARS-CoV-2 spike (25). Finally, this allows to avoid using real SARS-CoV-2 for long-term passaging experiments, which could potentially generate gain-of-function viral variants. After 20 passages in VeroE6-TMPRSS2, we titrated the evolved viruses in VeroE6-TMPRSS2 cells by visualizing virus-expressed GFP. We observed that a high proportion of viruses passaged at 33°C and 37°C induced small foci compared to the large foci showed by the founder virus (Fig. 2B). Large foci consisted of multinucleated syncytia, whereas small foci were formed by viruses with impaired cell–cell fusion capacity. Such viruses with low cell–cell fusogenicity were observed at a low frequency from passages 9–10 at 33°C and 37°C, and their proportion gradually increased until passage 15, when their average frequency plateaued at around 60% (Fig. 2C). In contrast, viruses evolved at 39°C all retained their large-foci phenotype, and no fusion-deficient viruses were observed at any passage (Fig. 2C). This demonstrates that temperature affects the phenotypic evolution of the SARS-CoV-2 spike, with hyperthermia (39°C) preventing changes in its cell–cell fusion activity.

### The evolution of SARS-CoV-2 spike variants is temperature-dependent

**Fig 3:**
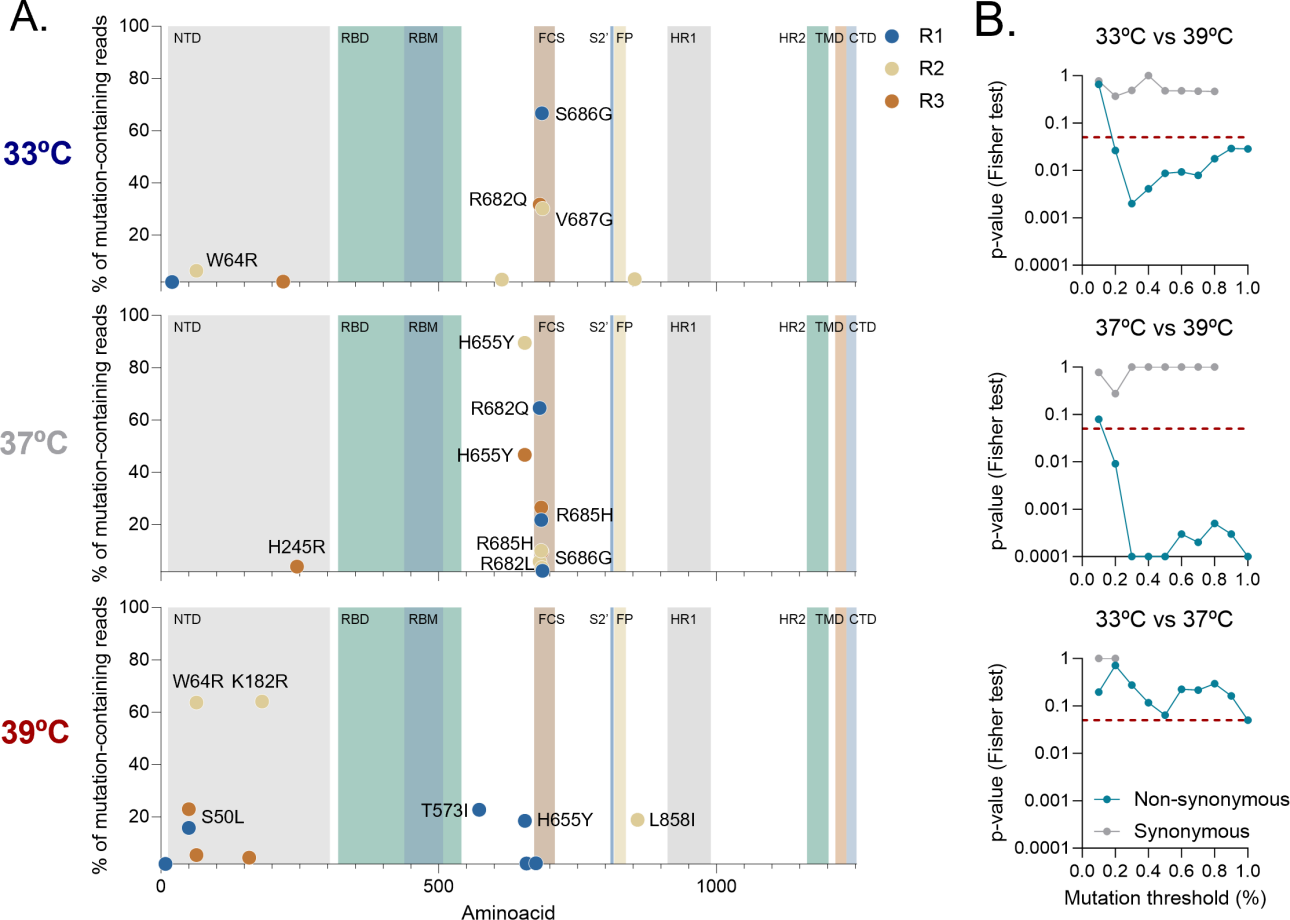
The SARS-CoV-2 spike sequence diversification is temperature-dependent. (A) Next-generation sequencing of viruses passaged 20 times at different temperatures. Non-synonymous mutations in the spike gene present at a frequency higher than 2% are represented. (**B)** The proportion of mutations in the region encompassed by residues 680 to 690 was compared between temperatures using a Fisher test, using different mutation frequency thresholds to include sequence variants (from 0.1 to 1%). This analysis was done separately for non-synonymous (blue) and synonymous (gray) mutations. The red dashed line indicates the statistical significance threshold (*P* = 0.05).

To understand the genetic basis of this temperature-dependent phenotypic evolution and to determine whether the spike genetic diversification is affected by temperature, we sequenced the evolved viral populations using Illumina (Fig. 3A). Most of the sequence variants that arose at a >2% frequency were not commonly observed in nature. An exception was H655Y, present in the Gamma and Omicron variants, which occurred in the three lineages evolved at 37°C and one of the 39°C replicates (Table S1). The T20N mutation, present in the Gamma variant, was also observed at a low frequency in all lineages. No high-frequency variant affected the receptor-binding domain (RBD), suggesting that ACE2 affinity is not a strong selective pressure in VeroE6-TMPRSS2. The S2 region was also very rarely affected, with only one high-frequency mutation (L858I) observed in only one lineage (39 °C R2). All lineages that evolved at 39°C had at least one N-terminal domain (NTD) mutation (S50L, W64R, and/or K182R) with a frequency >5%. NTD mutations were not observed as often in lineages evolved at 33°C and 37°C, suggesting that hyperthermia may promote NTD diversification.

More strikingly, in spikes evolved at 33°C or 37°C, there was a marked clustering of sequence variants in a region encompassed by residues 680 to 690, which contains the FCS. In contrast, this clustering was not observed in spikes evolved at 39°C. Specifically, all lineages evolved at 37°C and 33°C had at least one mutation in that region (R682Q, R682L, R685H, S686G, and/or V687G) at a frequency >5%, versus only one low-frequency (~0.1%) mutation (R682L) in the lineages evolved at 39°C. Some of these mutations appeared at a high frequency at both 33°C and 37°C (e.g., R682Q and S686G). To better analyze these differences, we compared the proportion of mutations falling at the FCS region versus the rest of the spike for lineages evolved at different temperatures (Fig. 3B). Non-synonymous mutations clustered significantly around the FCS in spikes evolved at 33°C or 37°C, but not at 39°C (Fisher’s exact test: *P* < 0.05). This association between the temperature used for evolution and FCS mutation clustering was significant for all variants found at a frequency >0.2%, whereas, below this threshold, differences were obscured, probably due to sequencing errors. We also found that the relative frequency of synonymous mutations at the FCS was similar between temperatures, suggesting that the aforementioned results were not due to an overall lower genetic diversification at higher temperatures. FCS mutations have previously been described to decrease syncytia formation (14, 15, 26) and thus explain the reduced fusogenicity of viruses evolved at 33°C and 37°C. Taken together, this shows that hyperthermia prevents the accumulation of sequence variants around the spike FCS, thereby preserving spike cell–cell fusogenicity, which is otherwise lost during passaging at 33°C or 37°C.

### Temperature-dependent effect of FCS mutations on spike performance

**Fig 4:**
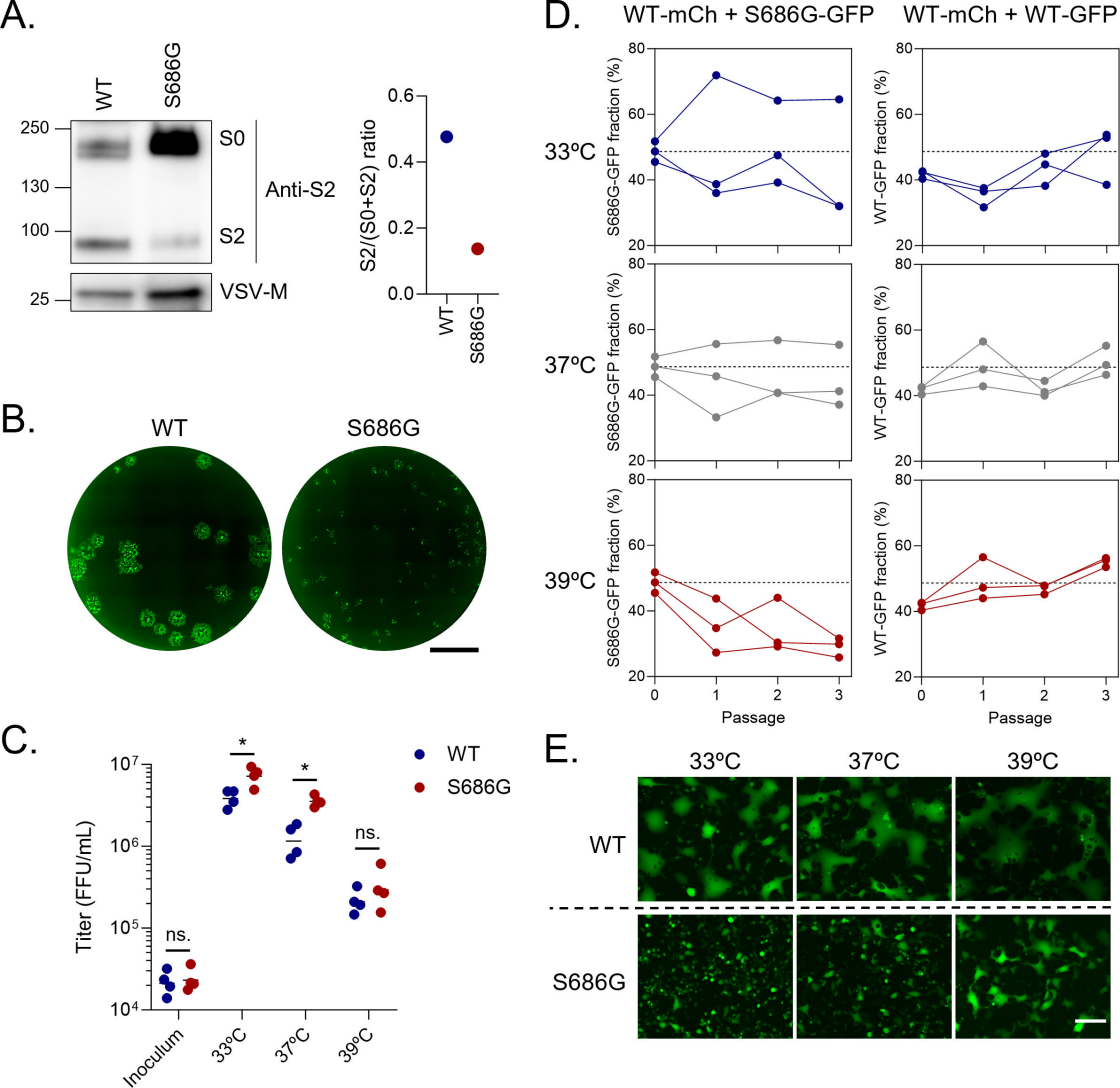
The effect of FCS mutations of spike-mediated cell–cell fusion and viral fitness is temperature-dependent. (A) Anti-spike S2 Western blot of rVSV-SC2 wild-type (WT) and S686G (left panel) and quantification of spike cleavage (right panel). (**B)** Foci morphology of rVSV-SC2 WT and S686G mutant. Scale bar: 3.4 mm. (**C)** VeroE6-TMPRSS2 were infected by WT or S686G rVSV-SC2 at different temperatures, and supernatants were harvested and titrated at 24 hpi. Each dot represents an independent experiment (*n* = 4), and the line indicates the mean. *P < 0.05; *ns*. not significant (paired log *t*-test). (**D)** Competition assay between rVSV-SC2-mCherry WT and rVSV-SC2-GFP S686G (left column) or WT (right column). VeroE6-TMPRSS2 were infected with a 1:1 mixture of both viruses, and three serial passages were performed. The proportion of GFP virus determined at 24 hpi after each passage is represented. Each line represents one independent replicate (*n* = 3). (**E)** Representative images of VeroE6-TMPRSS2 infected with rVSV-SC2 WT or S686G for 20 h at different temperatures. Scale bar: 200 µm.

To understand why FCS mutants evolved at 33°C and 37°C but not at 39°C, we generated an rVSV-SC2 carrying the S686G mutation, which disrupts the FCS, and measured the effects of this mutation on viral fitness. We chose the S686G as a prototype FCS mutation because it emerged in three different lineages evolved at two different temperatures. Moreover, it also appeared in another experimental evolution we performed in other cell lines (IGROV-1 and A549-ACE2-TMPRSS2) (27), suggesting that this mutation is a repeatable mechanism of FCS disruption during spike adaptation *in vitro*. We confirmed that S686G reduced spike cleavage by furin and spike-mediated cell–cell fusion, in agreement with previous findings (26) (Fig. 4A and B). This mutant thus mimics the phenotype observed in the experimental evolution and confirms that S686G is responsible, at least in part, for the reduced fusogenicity of the lineage in which it emerged (33°C R1 and 37°C R2-R3).

The rVSV-SC2 WT and S686G viruses were then used to infect VeroE6-TMPRSS2 at 33°C, 37°C, and 39°C using the same MOI as in the evolution experiment. Fitness was examined in two ways. First, we infected separate wells with each variant and determined the viral titer produced after 24 h (Fig. 4C). We found that the S686G mutant reached higher titers than the WT at 33°C (twofold; paired log *t*-test: *P* = 0.029) and 37°C (3.4-fold; paired log *t*-test: *P* = 0.025), but not at 39°C (paired log *t*-test: *P* = 0.31). The lack of an apparent benefit of this FCS mutation at 39°C may therefore explain why it was not selected at this temperature.

Second, we performed direct competition experiments by infecting each well with a 1:1 mixture of the rVSV-SC2 WT and rVSV-SC2 S686G viruses and propagating these mixed populations for three passages. The two competitors carried different fluorescent reporters in their genomes (mCherry and GFP, respectively), which allowed us to track their frequency by titration and counting of red versus green foci (Fig. 4D). At 33°C and 37°C, we failed to detect changes in the proportion of each variant after serial transfers (general linear model accounting for experimental block: *P* = 0.324 at 33°C; *P* = 0.174 at 37°C). However, at 39°C, the frequency of the S686G virus gradually decreased with passage number (48.6% in inoculum vs 29.1% at passage 3), the trend being highly significant (general linear model: *P* < 0.0001). In control assays, we verified that this change in frequency was not due to the different reporters expressed by the WT and S686G viruses (Fig. 4D). Thus, in competition, the S686G mutation was deleterious at 39°C.

Finally, we found that VeroE6-TMPRSS2 cells infected with the WT rVSV-SC2 fused massively at all temperatures (Fig. 4E). In contrast, infection with the S686G mutant induced cell–cell fusion in a highly temperature-dependent manner. At 33°C, almost no cell fusion was observed, whereas it was extensive at 39°C and close to that of the WT virus. Cells infected at 37°C showed an intermediate phenotype. We hence hypothesize that the fitness advantage of the S686G mutation at low temperatures may stem from reduced syncytia formation since cell–cell fusion can indeed be detrimental to long-term viral production due to premature cell death (12). This benefit would be lost at high temperatures, at which FCS disruption fails to prevent syncytia formation efficiently.

## DISCUSSION

We have investigated the effect of temperature on SARS-CoV-2 spike fusogenicity by measuring how it affects spike-mediated syncytia formation. In two different cell types (HEK293T and VeroE6-TMPRSS2), spike-mediated cell–cell fusion increased with temperature. A similar effect of temperature on syncytia formation has been described for HIV-1 (28), Sendai virus (28), and the SER paramyxovirus (29). However, this is not a general rule for syncytia-inducing viruses because cells infected with varicella zoster virus fused more at 33°C than at 37°C (30). The factors that determine the effect of temperature on virus-induced cell–cell fusion remain unclear and warrant further investigation. Temperature increases the fluidity of the plasma membrane, which could facilitate lipid mixing and membrane fusion. However, other processes could be affected by temperature and influence the fusogenicity of the SARS-CoV-2 spike. For example, spike processing is mediated by peptidases (e.g., furin, TMPRSS2, and cathepsins), the activity of which might be affected by temperature. One study predicted *in silico* that the affinity of the spike/furin interaction may increase with temperature (31). Temperature could also have a direct effect on spike conformation. A striking example of the modulation of viral structure by temperature is the transition of dengue virions from a smooth to rugged morphology when the temperature shifts from 28°C to 37°C, affecting the antigenicity and infectivity of viral particles (32, 33). Such a drastic effect of temperature on the structure of the SARS-CoV-2 spike has not been described, but low temperatures have been shown to promote the opening of the spike trimer, thereby increasing interactions with ACE2 (10, 34).

Syncytia have been observed in lung autopsy specimens from COVID-19 deceased patients (13, 35, 36). To our knowledge, syncytia have not been observed in other parts of the respiratory tract. Whether this is because this has not been investigated or because syncytia form specifically in the lung remains unclear. Our data suggest that the higher temperature found in the lower respiratory tract may favor syncytia formation. In addition, COVID-19-associated fever may increase spike-mediated cell–cell fusion. Given the proposed role of syncytia formation in viral pathogenesis (e.g., lung damage and inflammation), the effect of temperature on syncytia formation deserves further investigation in more relevant primary cell models or *in vivo*. The latter is complex in humans as histological analyses can only be performed in COVID-19 deceased patients. However, animal models (e.g., non-human primates and hamsters) infected with SARS-CoV-2 also show syncytia formation (37, 38) and could be used to study the tissue and temperature specificity of SARS-CoV-2 spike-mediated cell–cell fusion.

We used cell–cell fusion to measure spike fusogenicity. However, we did not determine whether high temperatures also increase the entry of SARS-CoV-2 virions. Increased membrane fluidity induced by high temperatures has previously been shown to increase the adsorption and entry of HIV-1 particles (39). Conversely, high membrane fluidity was detrimental to hepatitis C virus (HCV) entry (40). Therefore, as with cell–cell fusion, the effect of temperature on viral particle entry cannot be generalized to all viral species and should be further investigated for SARS-CoV-2. The effect of temperature on viral entry may act at different levels. For example, high temperatures inhibit influenza A virus infection by increasing endosomal pH (41). Since SARS-CoV-2 has been shown to require an acidic pH to infect cells (42), this relationship between temperature and pH may be important for SARS-CoV-2 entry. In addition, SARS-CoV-2 variants can use different entry pathways (plasma membrane vs endocytosis), suggesting that their susceptibility to temperature may differ.

It has been shown that FCS disruption is rapidly selected upon passage of SARS-CoV-2 into TMPRSS2-deficient cells (43–48), but it has been suggested that passaging the virus in TMPRSS2-expressing cells should avoid the emergence of these mutants (49). We therefore decided to perform our experimental evolution in VeroE6-TMPRSS2 cells to try to avoid this known bias. However, after 20 passages of rVSV-SC2 in VeroE6-TMPRSS2 at 33°C and 37°C, we repeatedly observed mutations around the FCS region that were associated with a reduced ability to form syncytia. This discrepancy with published results may be multi-factorial. First, to our knowledge, no study evaluated the effects of long-term passaging (>10 passages) of SARS-CoV-2 in VeroE6-TMPRSS2 cells. Second, the levels of TMPRSS2 expressed by our VeroE6-TMPRSS2 may differ from those in other studies. Finally, most studies describe the adaptation of real SARS-CoV-2 virus, whereas we used a recombinant VSV expressing the SARS-CoV-2 spike, a model that nevertheless captures relevant aspects of SARS-CoV-2 entry (22). We also note that the spike in our recombinant virus lacks the last 21 C-terminal amino acids of the S2 subunit, which has been shown to increase viral infectivity and syncytia formation (50).

The notion that syncytia formation is detrimental to viral fitness in cell cultures is widely supported by the systematic loss of the FCS and spike-induced cell–cell fusion reported here and in previous works ( 43–48). Given that spike-mediated cell–cell fusion was more extensive at 39°C, it may be expected that the selective pressure against fusion should also be highest at this temperature. In contrast, the virus did not lose the FCS during evolution at 39°C. The assays performed with the rVSV-SC2 and S686G viruses allowed us to explain these observations since we found that the FCS mutant induced syncytia formation at 39°C, as opposed to 33°C and 37°C. Hence, the fitness advantage of the FCS disruption at 33°C and 37°C was probably lost at 39°C because, at this temperature, cell–cell fusion was induced even by spikes with an impaired FCS. Whether the virus might find other ways to reduce cell–cell fusion at high temperatures is an open question that could be explored in the future by performing longer evolution experiments or passaging with alternating temperatures, which may better reflect what the virus faces during infection *in vivo*.

The fitness assays performed with the rVSV-SC2 WT and S686G viruses provided support to the temperature-dependent effect of FCS mutations on viral fitness. Specifically, the S686G mutation had a positive effect on viral titers at 33°C and 37°C, but not at 39°C. However, the results of the fitness measurements were not fully concordant between mono-infections and direct competition assays in which cultures were coinfected with both variants. The fitness of the S686G virus relative to the rVSV-SC2 WT was lower in coinfection than in mono-infection, such that the mutant was neutral at 33°C and 37°C, but deleterious at 39°C. This discrepancy could be explained in terms of virus–virus interactions. Cell–cell fusion decreases viral yields, but it can also allow rapid spread of the virus in the cell population since the viral release and entry stages of the infection cycle are bypassed. Such rapid spread of the WT syncytia-inducing virus might rapidly exhaust the population of non-infected cells, thus interfering with the growth of the FCS-deficient non-syncytia-inducing virus. Moreover, cells infected with the WT virus could fuse with S686G-infected cells, exerting a negative dominant effect, whereby the S686G mutation would fail to prevent syncytia formation. Consistent with these hypothetical scenarios, despite having a significantly positive effect on viral yield, FCS mutations did not reach fixation in our experimentally evolved populations, which contained a mixture of syncytia-inducing and fusion-deficient viruses. Future research may further address how virus–virus interactions modulate SARS-CoV-2 spike evolution and, more generally, syncytia induction by viruses.

In contrast to the fitness cost observed in cell cultures, syncytia induction might present some advantages for the virus *in vivo*, although this is a poorly understood topic. It has been suggested that syncytia formation may allow evasion of antibody-mediated neutralization (51). Syncytia may also be involved in viral pathogenesis. Indeed, one study showed that spike-mediated syncytia can engulf lymphocytes, inducing their intracellular death and thus representing a possible mechanism underlying COVID-19-associated lymphopenia (52). It has also been shown that syncytia are prone to premature cell death by apoptosis or pyroptosis (11, 53), which may contribute to excessive inflammation observed during SARS-CoV-2 infection, but also be detrimental to viral replication, as suggested by our results. It should also be noted that syncytia formation *in vivo* is likely to be less extensive than in our system since high viral titers, TMPRSS2 expression, and the absence of the E and M proteins facilitate syncytia formation (54). However, it is interesting to note that the Omicron variants, which rapidly replaced other variants, induce less syncytia formation than the ancestral strains (18–20). Whether syncytia formation is a selective pressure for SARS-CoV-2 and whether the virus has evolved to avoid premature cell death associated with extensive cell–cell fusion therefore deserves further investigation.

## MATERIALS AND METHODS

### Cell lines and cell culture

HEK293T-GFP1-10 and HEK293T-GFP11 cells were kindly provided by Olivier Schwartz (Institut Pasteur, Paris, France) and were cultured in the presence of 1 µg/mL of puromycin (Gibco). VeroE6-TMPRSS2 cells were grown in the presence of 500 µg/mL of G418 (Gibco). BHK-21 cells were obtained from the ATCC (ATCC CCL-10). BHK-G43 cells were maintained in the presence of hygromycin B (500 µg/mL) and zeocin (1 mg/mL). All cell lines were cultured in DMEM supplemented with 10% fetal bovine serum (FBS), 1% non-essential amino acids, 10 U/mL penicillin, 10 µg/mL streptomycin, and 250 ng/mL amphotericin B at 37°C and 5% CO_2_. Cell lines were regularly shown to be free of mycoplasma contamination by PCR.

### Viruses

The plasmid encoding the genome of VSV expressing the SARS-CoV-2 spike (Wuhan-Hu-1 strain; rVSV-SC2) deleted from its last 21 C-terminal amino acids instead of VSV-G (pVSVeGFP-∆G-Wu-S-∆Ct) was kindly provided by Dr. Ron Geller (CSIC, I2SysBio, Valencia, Spain). The rVSV-SC2 S686G plasmid was obtained through site-directed mutagenesis (Quickchange, Agilent) using a pair of completely overlapping primers (5′-CTCGGCGGGCACGTGGTGTAGCTAGTC-3′ and 5′-GACTAGCTACACCACGTGCCCGCCGAG-3′). Recovery of replication-competent recombinant VSV bearing the SARS-CoV-2 spike (wild-type or S686G) was performed as follows. Briefly, BHK-G43 cells (BHK-21 cells that express the VSV glycoprotein after mifepristone treatment (55), kindly provided by Dr. Gert Zimmer, Institute of Virology and Immunology (IVI), Mittelhäusern, Switzerland) were seeded at a density of 1.5 × 10^5^ cells/mL in DMEM 5% FBS without antibiotics in 12-well plates (1 mL per well). The following day, the viral genome pVSVeGFP-∆G-Wu-S-∆Ct was co-transfected with helper plasmids encoding VSV P (25 fmol), N (75 fmol), and L (25 fmol) proteins and the T7 RNA polymerase (50 fmol) using lipofectamine 3000 (Invitrogen) for 3 h at 37°C. Then, the medium was replaced with DMEM supplemented with 10% FBS with 10 nM mifepristone to induce VSV-G expression, and cells were incubated at 33°C for 36 h, followed by 48 h at 37°C. Supernatants from GFP-positive cells were harvested, clarified by centrifugation, at 10,000 × g for 10 min, and used to infect a fresh VSV-G-induced BHK-G43 p100 dish culture for amplification. Supernatants were harvested at 24–48 hours post-infection (hpi) and clarified in the same way. To clean viruses from VSV-G, non-G expressing cells were inoculated with the BHK-G43-amplified viruses, the inoculum was removed, and cells were washed five times with PBS and incubated in DMEM containing 2% FBS and 25% of an anti-VSV-G neutralizing monoclonal antibody obtained in-house from a mouse hybridoma cell line. Supernatants were harvested at 24–48 hpi, clarified by centrifugation, and stored at −80°C.

### GFP complementation cell–cell fusion assay

The SARS-CoV-2 HEK293T GFP-split assay was performed as previously described (11). Briefly, HEK293T-GFP1-10 and HEK293T-GFP11 were mixed at a 1:1 ratio (3 × 10^5^ cells of each cell type per well of a 96-well plate) and transfected with 50 ng of pcDNA3.1-SC2-spike (or an empty vector as a control) and 50 ng of pCG1-ACE2 plasmid using Lipofectamine 2000 (Invitrogen) following the manufacturer’s instructions. Cells were incubated overnight at 33°C and then shifted at different temperatures (33°C, 37°C, or 39°C) for the indicated time (0 h, 3 h, 6 h, or 8 h). Images were acquired in an IncuCyte SX5 Live-Cell Analysis System (Sartorius). The percentage of GFP-positive area and cell confluence was calculated with the IncuCyte analysis software, and the percentage of fusion was calculated as the ratio between GFP confluence and cell confluence.

### rVSV-SC2 experimental evolution

VeroE6-TMPRSS2 cells were plated at a 50% confluence in 6-well plates. The next day, cells were inoculated with 200 µL of virus dilution at an MOI of 0.06. Cells were placed in incubators at 33°C, 37°C, or 39°C and agitated every 20 min. After 2 h, 2 mL of DMEM supplemented with 2% FBS was added, and cells were incubated at different temperatures (33°C, 37°C, or 39°C). After 24 h, supernatants were harvested, cleared by centrifugation (2,000 × g for 10 min), aliquoted, and stored at −80°C. Between each passage, supernatants were titrated as described below. Two passages per week were performed until reaching 20 passages. Three independent replicate evolution lines were performed per temperature.

### Virus titration

VeroE6-TMPRSS2 were seeded in 24-well plates at a 100% confluence for 6–8 h before being inoculated for 1 h with serial dilution of virus (100 µL). Cells were then overlaid with 500 µL of DMEM containing 2% FBS and 0.5% agar. After overnight incubation at 37°C, plates were imaged in the IncuCyte SX5 Live-Cell Analysis System (Sartorius). GFP-positive foci were counted manually, and virus titers were calculated as focus-forming units (FFU) per mL.

### Next-generation sequencing

RNA from the initial viral stock (P0) and the evolved viruses (passage 20) was extracted using the QIAamp Viral RNA kit (Qiagen) following the manufacturer’s instructions. RNA was reverse-transcribed using SuperScript IV Reverse Transcriptase (Invitrogen) and a primer recognizing a sequence of the VSV genome upstream of the SARS-CoV-2 spike gene (5’- CTCGAACAACTAATATCCTGTC-3′). The SARS-CoV-2 spike gene was amplified by PCR using Phusion Hot Start II DNA polymerase (Thermo Scientific) and a set of primers recognizing sequences upstream and downstream of the spike gene (forward: 5’- CTCGAACAACTAATATCCTGTC-3′; reverse: 5′-GTTCTTACTATCCCACATCGAG-3′). PCR products were cleaned using the DNA Clean & Concentrator kit (Zymo Research) and analyzed by Illumina sequencing in an MiSeq machine with paired-end libraries (Novogene; read length: 2 × 250 bp). The quality of reads was analyzed with FastQC v0.11.5 (https://www.bioinformatics.babraham.ac.uk/projects/fastqc/). The first 15 and last 15 nucleotides of each read and adapters were removed using Cutadapt (https://cutadapt.readthedocs.io/en/stable/). Reads were then trimmed using the FASTQ quality filter (http://hannonlab.cshl.edu/fastx_toolkit/) and Prinseq-lite 0.20.4 by quality (>Q33), length (>100 nucleotides), and sequencing artifacts (duplications, Ns). The genome of the founder virus was used for mapping, and variant calling was performed with FreeBayes (56). The spike sequencing coverage was homogenous across samples and averaged 7,956 ± 196.

### Western blotting

A 1 mL volume of the supernatant containing rVSV-SC2 was pelleted by centrifugation at 30,000 × g for 2 h and lysed in 30 µL of NP-40 lysis buffer (Invitrogen) for 30 min on ice. Viral lysates were mixed with 4× Laemlli buffer (Bio-Rad) supplemented with 10% β-mercaptoethanol and denaturated at 95°C for 5 min. Proteins were separated by SDS-PAGE on a 4%–20% Mini-PROTEAN TGX Gel (Bio-Rad) and transferred onto a 0.45-µm PVDF membrane (Thermo Scientific). Membranes were blocked for 1 h at room temperature in TBS-T (20 mM tris, 150 mM NaCl, 0.1% Tween-20, pH 7.5) supplemented with 3% bovine serum albumin (BSA; Sigma). Membranes were then incubated for 1 h at RT with the following primary antibodies: mouse anti-SARS-CoV-2 S2 (dilution 1:2,000, clone 1A9, GeneTex) and mouse anti-VSV-M (dilution 1:1,000, clone 23H12, Kerafast). Membranes were washed three times with TBS-T and incubated 1 h at RT with an HRP-conjugated anti-mouse secondary antibody (dilution 1:50,000, G-21040, Invitrogen). After washing three times in TBS-T, the signal was revealed with SuperSignal West Pico PLUS (Thermo Scientific) following the manufacturer’s instructions. Images were acquired on an ImageQuant LAS 500 (GE Healthcare) and analyzed with Fiji software.

### Fitness assays

VeroE6-TMPRSS2 cells were plated at a 50% confluence in 12-well plates. The next day, cells were inoculated with 150 µL of virus dilution at an MOI of 0.06. Cells were incubated at 33°C, 37°C, or 39°C and agitated every 20 min. After 2 h, 1 mL of DMEM containing 2% FBS was added, and cells were incubated at different temperatures (33°C, 37°C, or 39°C). After 24 h, supernatants were harvested, clarified by centrifugation (2,000 × g for 10 min), aliquoted, and stored at −80°C until titration as described above.

### Competition assays

VeroE6-TMPRSS2 cells were plated at a 50% confluence in 12-well plates. The next day, cells were inoculated with 150 µL of a 1:1 mixture of rVSV-SC2-mCherry WT and rVSV-SC2-GFP S686G (total MOI = 0.06). A control condition consisting of a 1:1 mixture of rVSV-SC2-mCherry WT and rVSV-SC2-GFP WT (total MOI = 0.06) was included to ensure the observed differences were not due to the expression of GFP vs mCherry. Cells were placed in incubators at 33°C, 37°C, or 39°C and agitated every 20 min. After 2 h, 1 mL of DMEM with 2% FBS was added, and cells were incubated at different temperatures (33°C, 37°C, or 39°C). Twenty-four hours later, supernatants were harvested, cleared by centrifugation (2,000 × g for 10 min), aliquoted, and stored at −80°C. Supernatants were then titrated as described above with the difference that both GFP^+^ and mCherry^+^ foci were counted. Supernatants were then used to initiate a new passage by adjusting the total MOI to 0.06. Three passages were performed in total, and the percentage of GFP foci after each passage was quantified. Three independent replicates were performed at each temperature.

### Statistics

Statistics were performed in GraphPad Prism v10 or SPSS v28. All details about statistical tests can be found in the figure legends or in the main text.

## SUPPLEMENTAL MATERIAL

Table S1 mbio.03360-23-s0001.xlsx Mutation frequencies.

ASM does not own the copyrights to Supplemental Material that may be linked to, or accessed through, an article. The authors have granted ASM a non-exclusive, world-wide license to publish the Supplemental Material files. Please contact the corresponding author directly for reuse.

## References

[1] Liu J, Li Y, Liu Q, Yao Q, Wang X, Zhang H, Chen R, Ren L, Min J, Deng F, Yan B, Liu L, Hu Z, Wang M, Zhou Y. 2021. SARS-Cov-2 cell tropism and multiorgan infection. Cell Discov 7:17. doi:10.1038/s41421-021-00249-2 33758165 PMC7987126

[2] Ahn JH, Kim J, Hong SP, Choi SY, Yang MJ, Ju YS, Kim YT, Kim HM, Rahman MDT, Chung MK, Hong SD, Bae H, Lee C-S, Koh GY. 2021. Nasal ciliated cells are primary targets for SARS-Cov-2 replication in the early stage of COVID-19. J Clin Invest 131:e148517. doi:10.1172/JCI148517 34003804 PMC8245175

[3] Hui KPY, Ng K-C, Ho JCW, Yeung H-W, Ching RHH, Gu H, Chung JCK, Chow VLY, Sit K-Y, Hsin MKY, Au TWK, Poon LLM, Peiris M, Nicholls JM, Chan MCW. 2022. Replication of SARS-Cov-2 Omicron BA.2 variant in ex vivo cultures of the human upper and lower respiratory tract. EBioMedicine 83:104232. doi:10.1016/j.ebiom.2022.104232 35988466 PMC9387351

[4] Hu B, Guo H, Zhou P, Shi ZL. 2021. Characteristics of SARS-Cov-2 and COVID-19. Nat Rev Microbiol 19:141–154. doi:10.1038/s41579-020-00459-7 33024307 PMC7537588

[5] V’kovski P, Gultom M, Kelly JN, Steiner S, Russeil J, Mangeat B, Cora E, Pezoldt J, Holwerda M, Kratzel A, Laloli L, Wider M, Portmann J, Tran T, Ebert N, Stalder H, Hartmann R, Gardeux V, Alpern D, Deplancke B, Thiel V, Dijkman R. 2021. Disparate temperature-dependent virus–host dynamics for SARS-CoV-2 and SARS-CoV in the human respiratory epithelium. PLoS Biol 19:e3001158. doi:10.1371/journal.pbio.3001158 33780434 PMC8032198

[6] Otter CJ, Fausto A, Tan LH, Khosla AS, Cohen NA, Weiss SR. 2023. Infection of primary nasal epithelial cells differentiates among lethal and seasonal human coronaviruses. Proc Natl Acad Sci USA 120:e2218083120. doi:10.1073/pnas.2218083120 37023127 PMC10104492

[7] Herder V, Dee K, Wojtus JK, Epifano I, Goldfarb D, Rozario C, Gu Q, Da Silva Filipe A, Nomikou K, Nichols J, Jarrett RF, Stevenson A, McFarlane S, Stewart ME, Szemiel AM, Pinto RM, Masdefiol Garriga A, Davis C, Allan J, Graham SV, Murcia PR, Boutell C. 2021. Elevated temperature inhibits SARS-Cov-2 replication in respiratory epithelium independently of IFN-mediated innate immune defenses. PLoS Biol 19:e3001065. doi:10.1371/journal.pbio.3001065 34932557 PMC8765667

[8] Bisht K, Te Velthuis AJW. 2022. Decoding the role of temperature in RNA virus infections. mBio 13:e0202122. doi:10.1128/mbio.02021-22 35980031 PMC9600459

[9] Gong SY, Ding S, Benlarbi M, Chen Y, Vézina D, Marchitto L, Beaudoin-Bussières G, Goyette G, Bourassa C, Bo Y, Medjahed H, Levade I, Pazgier M, Côté M, Richard J, Prévost J, Finzi A. 2022. Temperature influences the interaction between SARS-CoV-2 spike from Omicron subvariants and human ACE2. Viruses 14:2178. doi:10.3390/v14102178 36298733 PMC9607596

[10] Prévost J, Richard J, Gasser R, Ding S, Fage C, Anand SP, Adam D, Gupta Vergara N, Tauzin A, Benlarbi M, Gong SY, Goyette G, Privé A, Moreira S, Charest H, Roger M, Mothes W, Pazgier M, Brochiero E, Boivin G, Abrams CF, Schön A, Finzi A. 2021. Impact of temperature on the affinity of SARS-CoV-2 spike glycoprotein for host ACE2. J Biol Chem 297:101151. doi:10.1016/j.jbc.2021.101151 34478710 PMC8406544

[11] Buchrieser J, Dufloo J, Hubert M, Monel B, Planas D, Rajah MM, Planchais C, Porrot F, Guivel-Benhassine F, Van der Werf S, Casartelli N, Mouquet H, Bruel T, Schwartz O. 2021. Syncytia formation by SARS‐CoV‐2‐infected cells. EMBO J 40:e107405. doi:10.15252/embj.2020107405 33522642 PMC7849166

[12] Rajah MM, Bernier A, Buchrieser J, Schwartz O. 2022. The mechanism and consequences of SARS-CoV-2 spike-mediated fusion and syncytia formation. J Mol Biol 434:167280. doi:10.1016/j.jmb.2021.167280 34606831 PMC8485708

[13] Braga L, Ali H, Secco I, Chiavacci E, Neves G, Goldhill D, Penn R, Jimenez-Guardeño JM, Ortega-Prieto AM, Bussani R, Cannatà A, Rizzari G, Collesi C, Schneider E, Arosio D, Shah AM, Barclay WS, Malim MH, Burrone J, Giacca M. 2021. Drugs that inhibit TMEM16 proteins block SARS-CoV-2 spike-induced syncytia. Nature 594:88–93. doi:10.1038/s41586-021-03491-6 33827113 PMC7611055

[14] Lavie M, Dubuisson J, Belouzard S. 2022. SARS-CoV-2 spike furin cleavage site and S2′ basic residues modulate the entry process in a host cell-dependent manner. J Virol 96:e0047422. doi:10.1128/jvi.00474-22 35678602 PMC9278140

[15] Papa G, Mallery DL, Albecka A, Welch LG, Cattin-Ortolá J, Luptak J, Paul D, McMahon HT, Goodfellow IG, Carter A, Munro S, James LC. 2021. Furin cleavage of SARS-CoV-2 spike promotes but is not essential for infection and cell-cell fusion. PLoS Pathog 17:e1009246. doi:10.1371/journal.ppat.1009246 33493182 PMC7861537

[16] Harvey WT, Carabelli AM, Jackson B, Gupta RK, Thomson EC, Harrison EM, Ludden C, Reeve R, Rambaut A, COVID-19 Genomics UK (COG-UK) Consortium, Peacock SJ, Robertson DL. 2021. SARS-Cov-2 variants, spike mutations and immune escape. Nat Rev Microbiol 19:409–424. doi:10.1038/s41579-021-00573-0 34075212 PMC8167834

[17] Rajah MM, Hubert M, Bishop E, Saunders N, Robinot R, Grzelak L, Planas D, Dufloo J, Gellenoncourt S, Bongers A, Zivaljic M, Planchais C, Guivel-Benhassine F, Porrot F, Mouquet H, Chakrabarti LA, Buchrieser J, Schwartz O. 2021. SARS‐CoV‐2 alpha, beta, and delta variants display enhanced spike‐mediated syncytia formation. EMBO J 40:e108944. doi:10.15252/embj.2021108944 34601723 PMC8646911

[18] Meng B, Abdullahi A, Ferreira IATM, Goonawardane N, Saito A, Kimura I, Yamasoba D, Gerber PP, Fatihi S, Rathore S, et al.. 2022. Altered TMPRSS2 usage by SARS-CoV-2 Omicron impacts infectivity and fusogenicity. Nature 603:706–714. doi:10.1038/s41586-022-04474-x 35104837 PMC8942856

[19] Yamasoba D, Kimura I, Nasser H, Morioka Y, Nao N, Ito J, Uriu K, Tsuda M, Zahradnik J, Shirakawa K, et al.. 2022. Virological characteristics of the SARS-CoV-2 Omicron BA.2 spike. Cell 185:2103–2115. doi:10.1016/j.cell.2022.04.035 35568035 PMC9057982

[20] Suzuki R, Yamasoba D, Kimura I, Wang L, Kishimoto M, Ito J, Morioka Y, Nao N, Nasser H, Uriu K, et al.. 2022. Attenuated Fusogenicity and Pathogenicity of SARS-Cov-2 Omicron variant. Nature 603:700–705. doi:10.1038/s41586-022-04462-1 35104835 PMC8942852

[21] Muramoto Y, Takahashi S, Halfmann PJ, Gotoh S, Noda T, Kawaoka Y. 2023. Replicative capacity of SARS-Cov-2 Omicron variants BA.5 and BQ.1.1 at elevated temperatures. Lancet Microbe 4:e486. doi:10.1016/S2666-5247(23)00100-3 37105204 PMC10124997

[22] Dieterle ME, Haslwanter D, Bortz RH, Wirchnianski AS, Lasso G, Vergnolle O, Abbasi SA, Fels JM, Laudermilch E, Florez C, Mengotto A, Kimmel D, Malonis RJ, Georgiev G, Quiroz J, Barnhill J, Pirofski L-A, Daily JP, Dye JM, Lai JR, Herbert AS, Chandran K, Jangra RK. 2020. A replication-competent vesicular stomatitis virus for studies of SARS-CoV-2 spike-mediated cell entry and its inhibition. Cell Host Microbe 28:486–496. doi:10.1016/j.chom.2020.06.020 32738193 PMC7332447

[23] Schmidt F, Weisblum Y, Muecksch F, Hoffmann H-H, Michailidis E, Lorenzi JCC, Mendoza P, Rutkowska M, Bednarski E, Gaebler C, Agudelo M, Cho A, Wang Z, Gazumyan A, Cipolla M, Caskey M, Robbiani DF, Nussenzweig MC, Rice CM, Hatziioannou T, Bieniasz PD. 2020. Measuring SARS-CoV-2 neutralizing antibody activity using pseudotyped and chimeric viruses. J Exp Med 217:e20201181. doi:10.1084/jem.20201181 32692348 PMC7372514

[24] Case JB, Rothlauf PW, Chen RE, Liu Z, Zhao H, Kim AS, Bloyet L-M, Zeng Q, Tahan S, Droit L, Ilagan M, Tartell MA, Amarasinghe G, Henderson JP, Miersch S, Ustav M, Sidhu S, Virgin HW, Wang D, Ding S, Corti D, Theel ES, Fremont DH, Diamond MS, Whelan SPJ. 2020. Neutralizing antibody and soluble ACE2 inhibition of a replication-competent VSV-SARS-CoV-2 and a clinical isolate of SARS-CoV-2. Cell Host Microbe 28:475–485. doi:10.1016/j.chom.2020.06.021 32735849 PMC7332453

[25] Weisblum Y, Schmidt F, Zhang F, DaSilva J, Poston D, Lorenzi JC, Muecksch F, Rutkowska M, Hoffmann H-H, Michailidis E, Gaebler C, Agudelo M, Cho A, Wang Z, Gazumyan A, Cipolla M, Luchsinger L, Hillyer CD, Caskey M, Robbiani DF, Rice CM, Nussenzweig MC, Hatziioannou T, Bieniasz PD. 2020. Escape from neutralizing antibodies 1 by SARS-CoV-2 spike protein variants. Elife 9:e61312. doi:10.7554/eLife.61312 33112236 PMC7723407

[26] Arora P, Sidarovich A, Graichen L, Hörnich B, Hahn A, Hoffmann M, Pöhlmann S. 2022. Functional analysis of polymorphisms at the S1/S2 site of SARS-CoV-2 spike protein. PLoS One 17:e0265453. doi:10.1371/journal.pone.0265453 35333910 PMC8956166

[27] Carrascosa-Sàez M, Marqués M-C, Geller R, Elena SF, Rahmeh A, Dufloo J, Sanjuán R, The IBV-Covid19-Pipeline. 2023. Cell type-specific adaptation of the SARS-CoV-2 spike. bioRxiv. doi:10.1101/2023.12.20.572504 PMC11110937 38779130

[28] Kinchington D, Barker W, Galpin S, Apostolov K. 1992. Temperature enhancement of syncytium formation by HIV and sendai virus. J Med Virol 36:44–48. doi:10.1002/jmv.1890360109 1315369

[29] Seth S, Vincent A, Compans RW. 2003. Activation of fusion by the SER virus F protein: a low-pH-dependent paramyxovirus entry process. J Virol 77:6520–6527. doi:10.1128/jvi.77.11.6520-6527.2003 12743308 PMC155032

[30] Cole NL, Grose C. 2003. Membrane fusion mediated by herpesvirus glycoproteins: the paradigm of varicella-zoster virus. Rev Med Virol 13:207–222. doi:10.1002/rmv.377 12820183

[31] Mohammad A, Alshawaf E, Marafie SK, Abu-Farha M, Abubaker J, Al-Mulla F. 2021. Higher binding affinity of furin for SARS-CoV-2 spike (S) protein D614G mutant could be associated with higher SARS-CoV-2 infectivity. Int J Infect Dis 103:611–616. doi:10.1016/j.ijid.2020.10.033 33075532 PMC7567667

[32] Zhang X, Sheng J, Plevka P, Kuhn RJ, Diamond MS, Rossmann MG. 2013. Dengue structure differs at the temperatures of its human and mosquito hosts. Proc Natl Acad Sci USA 110:6795–6799. doi:10.1073/pnas.1304300110 23569243 PMC3637732

[33] Fibriansah G, Ng T-S, Kostyuchenko VA, Lee J, Lee S, Wang J, Lok S-M. 2013. Structural changes in dengue virus when exposed to a temperature of 37°C. J Virol 87:7585–7592. doi:10.1128/JVI.00757-13 23637405 PMC3700303

[34] Costello SM, Shoemaker SR, Hobbs HT, Nguyen AW, Hsieh C-L, Maynard JA, McLellan JS, Pak JE, Marqusee S. 2022. The SARS-CoV-2 spike reversibly samples an open-trimer conformation exposing novel epitopes. Nat Struct Mol Biol 29:229–238. doi:10.1038/s41594-022-00735-5 35236990 PMC9007726

[35] Bussani R, Schneider E, Zentilin L, Collesi C, Ali H, Braga L, Volpe MC, Colliva A, Zanconati F, Berlot G, Silvestri F, Zacchigna S, Giacca M. 2020. Persistence of viral RNA, pneumocyte Syncytia and thrombosis are hallmarks of advanced COVID-19 pathology. EBioMedicine 61:103104. doi:10.1016/j.ebiom.2020.103104 33158808 PMC7677597

[36] Luo W-R, Yu H, Gou J-Z, Li X-X, Sun Y, Li J-X, He J-X, Liu L. 2020. Histopathologic findings in the explant lungs of a patient with COVID-19 treated with bilateral orthotopic lung transplant. Transplantation 104:e329–e331. doi:10.1097/TP.0000000000003412 33122591

[37] Castellan M, Zamperin G, Franzoni G, Foiani G, Zorzan M, Drzewnioková P, Mancin M, Brian I, Bortolami A, Pagliari M, Oggiano A, Vascellari M, Panzarin V, Crovella S, Monne I, Terregino C, De Benedictis P, Leopardi S. 2023. Host response of syrian Hamster to SARS-CoV-2 infection including differences with humans and between sexes. Viruses 15:428. doi:10.3390/v15020428 36851642 PMC9960357

[38] Rockx B, Kuiken T, Herfst S, Bestebroer T, Lamers MM, Oude Munnink BB, de Meulder D, van Amerongen G, van den Brand J, Okba NMA, Schipper D, van Run P, Leijten L, Sikkema R, Verschoor E, Verstrepen B, Bogers W, Langermans J, Drosten C, Fentener van Vlissingen M, Fouchier R, de Swart R, Koopmans M, Haagmans BL. 2020. Comparative pathogenesis of COVID-19, MERS, and SARS in a nonhuman primate model. Science 368:1012–1015. doi:10.1126/science.abb7314 32303590 PMC7164679

[39] Harada S, Akaike T, Yusa K, Maeda Y. 2004. Adsorption and infectivity of human immunodeficiency virus type 1 are modified the fluidity of the plasma membrane for multiple-site binding. Microbiol Immunol 48:347–355. doi:10.1111/j.1348-0421.2004.tb03516.x 15107546

[40] Chamoun-Emanuelli AM, Pecheur E-I, Simeon RL, Huang D, Cremer PS, Chen Z. 2013. Phenothiazines inhibit hepatitis C virus entry, likely by increasing the fluidity of cholesterol-rich membranes. Antimicrob Agents Chemother 57:2571–2581. doi:10.1128/AAC.02593-12 23529728 PMC3716126

[41] Yamaya M, Nishimura H, Lusamba Kalonji N, Deng X, Momma H, Shimotai Y, Nagatomi R. 2019. Effects of high temperature on pandemic and seasonal human influenza viral replication and infection-induced damage in primary human tracheal epithelial cell cultures. Heliyon 5:e01149. doi:10.1016/j.heliyon.2019.e01149 30839917 PMC6365403

[42] Kreutzberger AJB, Sanyal A, Saminathan A, Bloyet L-M, Stumpf S, Liu Z, Ojha R, Patjas MT, Geneid A, Scanavachi G, Doyle CA, Somerville E, Correia RBDC, Di Caprio G, Toppila-Salmi S, Mäkitie A, Kiessling V, Vapalahti O, Whelan SPJ, Balistreri G, Kirchhausen T. 2022. SARS-Cov-2 requires acidic pH to infect cells. Proc Natl Acad Sci U S A 119:e2209514119. doi:10.1073/pnas.2209514119 36048924 PMC9499588

[43] Ogando NS, Dalebout TJ, Zevenhoven-Dobbe JC, Limpens R, van der Meer Y, Caly L, Druce J, de Vries JJC, Kikkert M, Bárcena M, Sidorov I, Snijder EJ. 2020. SARS-Coronavirus-2 replication in vero E6 cells: replication kinetics, rapid adaptation and cytopathology. J Gen Virol 101:925–940. doi:10.1099/jgv.0.001453 32568027 PMC7654748

[44] Chaudhry MZ, Eschke K, Hoffmann M, Grashoff M, Abassi L, Kim Y, Brunotte L, Ludwig S, Kröger A, Klawonn F, Pöhlmann SH, Cicin-Sain L. 2022. Rapid SARS-CoV-2 adaptation to available cellular proteases. J Virol 96:e0218621. doi:10.1128/jvi.02186-21 35019723 PMC8906416

[45] Ogando NS, Dalebout TJ, Zevenhoven-Dobbe JC, Limpens R, van der Meer Y, Caly L, Druce J, de Vries JJC, Kikkert M, Bárcena M, Sidorov I, Snijder EJ. 2020. SARS-coronavirus-2 replication in vero E6 cells: replication kinetics, rapid adaptation and cytopathology. J Gen Virol 101:925–940. doi:10.1099/jgv.0.001453 32568027 PMC7654748

[46] Lau S-Y, Wang P, Mok B-Y, Zhang AJ, Chu H, Lee A-Y, Deng S, Chen P, Chan K-H, Song W, Chen Z, To K-W, Chan J-W, Yuen K-Y, Chen H. 2020. Attenuated SARS-CoV-2 variants with deletions at the S1/S2 junction. Emerg Microbes Infect 9:837–842. doi:10.1080/22221751.2020.1756700 32301390 PMC7241555

[47] Liu Z, Zheng H, Lin H, Li M, Yuan R, Peng J, Xiong Q, Sun J, Li B, Wu J, Yi L, Peng X, Zhang H, Zhang W, Hulswit RJG, Loman N, Rambaut A, Ke C, Bowden TA, Pybus OG, Lu J. 2020. Identification of common deletions in the spike protein of severe acute respiratory syndrome coronavirus 2. J Virol 94:e00790-20. doi:10.1128/JVI.00790-20 32571797 PMC7431800

[48] Davidson AD, Williamson MK, Lewis S, Shoemark D, Carroll MW, Heesom KJ, Zambon M, Ellis J, Lewis PA, Hiscox JA, Matthews DA. 2020. Characterisation of the transcriptome and proteome of SARS-CoV-2 reveals a cell passage induced in-frame deletion of the furin-like cleavage site from the spike glycoprotein. Genome Med 12:68. doi:10.1186/s13073-020-00763-0 32723359 PMC7386171

[49] Lamers MM, Mykytyn AZ, Breugem TI, Wang Y, Wu DC, Riesebosch S, van den Doel PB, Schipper D, Bestebroer T, Wu NC, Haagmans BL. 2021. Human airway cells prevent SARS-CoV-2 multibasic cleavage site cell culture adaptation. Elife 10:e66815. doi:10.7554/eLife.66815 33835028 PMC8131099

[50] Yu J, Li Z, He X, Gebre MS, Bondzie EA, Wan H, Jacob-Dolan C, Martinez DR, Nkolola JP, Baric RS, Barouch DH, Silvestri G. 2021. Deletion of the SARS-CoV-2 spike cytoplasmic tail increases infectivity in pseudovirus neutralization assays. J Virol 95:e00044-21. doi:10.1128/JVI.00044-21 33727331 PMC8139703

[51] Zeng C, Evans JP, King T, Zheng Y-M, Oltz EM, Whelan SPJ, Saif LJ, Peeples ME, Liu S-L. 2022. SARS-CoV-2 spreads through cell-to-cell transmission. Proc Natl Acad Sci USA 119:e2111400119. doi:10.1073/pnas.2111400119 34937699 PMC8740724

[52] Zhang Z, Zheng Y, Niu Z, Zhang B, Wang C, Yao X, Peng H, Franca DN, Wang Y, Zhu Y, et al.. 2021. SARS-CoV-2 spike protein dictates syncytium-mediated lymphocyte elimination. Cell Death Differ 28:2765–2777. doi:10.1038/s41418-021-00782-3 33879858 PMC8056997

[53] Ma H, Zhu Z, Lin H, Wang S, Zhang P, Li Y, Li L, Wang J, Zhao Y, Han J. 2021. Pyroptosis of syncytia formed by fusion of SARS-CoV-2 spike and ACE2-expressing cells. Cell Discov 7:73. doi:10.1038/s41421-021-00310-0 34429403 PMC8384103

[54] Boson B, Legros V, Zhou B, Siret E, Mathieu C, Cosset F-L, Lavillette D, Denolly S. 2021. The SARS-CoV-2 envelope and membrane proteins modulate maturation and retention of the spike protein, allowing assembly of virus-like particles. J Biol Chem 296:100111. doi:10.1074/jbc.RA120.016175 33229438 PMC7833635

[55] Hanika A, Larisch B, Steinmann E, Schwegmann-Weßels C, Herrler G, Zimmer G. 2005. Use of influenza C virus glycoprotein HEF for generation of vesicular stomatitis virus pseudotypes. J Gen Virol 86:1455–1465. doi:10.1099/vir.0.80788-0 15831958

[56] Garrison E, Marth G. 2012. Haplotype-based variant detection from short-read sequencing. arXiv Preprint

